# A mouse model of MEPAN demonstrates a role for mitochondrial fatty acid synthesis in iron–sulfur cluster and supercomplex formation

**DOI:** 10.1073/pnas.2506761122

**Published:** 2025-09-29

**Authors:** Deborah G. Murdock, Kevin A. Janssen, Kierstin Keller, Katherine L. Mitchell, Maina Beauplan, William T. O’Brien, Lia D’Alessandro, Jeffrey A. Haltom, Douglas C. Wallace

**Affiliations:** ^a^Center for Mitochondrial and Epigenomic Medicine, Children’s Hospital of Philadelphia, Philadelphia, PA 19104; ^b^Department of Pediatrics, Division of Human Genetics, The Children’s Hospital of Philadelphia, Perelman School of Medicine, University of Pennsylvania, Philadelphia, PA 19104; ^c^Neurobehavior Testing Core, University of Pennsylvania, Philadelphia, PA 19104

**Keywords:** mitochondrial disease, mouse model, iron, genetics, mitochondrial fatty acid synthesis

## Abstract

MEPAN (Mitochondrial Enoyl CoA Reductase Protein-Associated Neurodegeneration) is an early-onset movement disorder characterized by ataxia, dysarthria, and optic atrophy. Here, we report the creation of a mouse model of MEPAN with patient-similar compound heterozygous mutations in the *Mecr* gene. The MEPAN mouse recapitulates the major hallmarks of MEPAN, including a movement disorder, optic neuropathy, defects in protein lipoylation, and reduced mitochondrial oxidative phosphorylation in the brain. MECR catalyzes the last step in mitochondrial fatty acid synthesis (mtFASII), and the mechanism by which loss of mtFASII leads to neurological disease is unknown. LC–MS/MS-based proteomic analysis of *Mecr* mutant cerebella identified loss of subunits of complex I of oxidative phosphorylation (OXPHOS) and subunits of the iron–sulfur cluster assembly (ISC) complex. Native gels revealed altered OXPHOS complex and supercomplex formation and changes in binding of the acyl carrier protein (ACP) to mitochondrial complexes. These results demonstrate that MECR plays a key role in the acylation of ACP which is necessary for ACP-LYRM-mediated supercomplex modulation and ISC biogenesis and suggest unique pathways for therapeutics.

## Significance

The mitochondrial fatty acid synthesis pathway is necessary for mitochondrial ATP production, but its mechanism is unknown. Individuals with mutations in *MECR*, encoding the last step in mtFASII, have early-onset neurological dysfunction. We describe characterization of a mouse model of MEPAN and the mechanism by which mtFASII causes neurological dysfunction. Dysfunctional MECR results in loss of iron–sulfur center cluster biogenesis complexes and altered formation of complexes and supercomplexes of OXPHOS through the loss of acylation of ACP and its interactions with LYRM proteins. These results offer therapeutic targets for treatment of MEPAN and mitochondrial modulation in general.

The identification of individuals with mutations in the *MECR* gene provides a window into the role of mitochondrial fatty acid synthesis (mtFASII) in mitochondrial health in mammalian systems. Mutations in *MECR* have been identified in individuals with a constellation of phenotypes called MEPAN (Mitochondrial Enoyl CoA Reductase Protein-Associated Neurodegeneration) (1–5). These patients typically present with an early-onset movement disorder, ataxia, dysarthria, and optic atrophy. MECR catalyzes the last step in a four-step spiral of reactions that synthesize fatty acids in mitochondria (6, 7). Complete deletion of *MECR* results in loss of mitochondrial respiration and rudimentary mitochondrial remnants in yeast (8) and embryonic lethality in mice (9). Across species, mtFASII disruption results in loss of mitochondrial respiration with reduced activity of multiple complexes of oxidative phosphorylation (OXPHOS) and defective lipoylation of pyruvate dehydrogenase, α-ketoglutarate dehydrogenase, branched chain alpha ketoacid dehydrogenase, and glycine cleavage system protein H (GCSH) (2, 10, 11).

While cytosolic fatty acid synthesis (FASI) uses coenzyme A (CoA) as the fatty acid carrier, mtFASII relies on the scaffold protein acyl carrier protein (ACP). The posttranslational modification of ACP with a 4′-phosphopantetheine (4′-PP) prosthetic group on serine 112 is necessary for attachment of the growing acyl chain and for interaction with LYR (Leucine-Tyrosine-Arginine) motif-containing proteins. Mitochondrial ACP was originally identified as a component of complex I (NADH:ubiquinone oxidoreductase) (12), where it resides in two distinct locations: at the junction between the Q module and the ND2 module and at the end of the ND5 module (13). ACP is also a resident protein of the iron–sulfur cluster (ISC) assembly complex in mammalian cells (14–16). The ISC complex is necessary for the assembly of iron–sulfur (FeS) clusters that act as catalytic sites in several enzymes, including the first three complexes of the electron transport chain, aconitase, and lipoic acid synthase. The ISC complex consists of cysteine desulfurase (NFS), ISCU, frataxin (FTXN), ferredoxin (FTX2), ISD11 (LYRM4), and ACP. ACP interacts with ISD11 (LYRM4) through the pantothenate group of ACP, and the fatty acid attached to ACP is flipped into the hydrophobic core of ISD11 suggesting a role for mtFASII-derived fatty acids in the assembly of the complex (17–19). ISD11 belongs to a larger family of small mitochondrial proteins with a leucine tyrosine arginine motif (LYRM) that are important for the assembly of several mitochondrial protein complexes through their interaction with ACP (16, 20). LYRM proteins link ACP to the biogenesis of complex I, II, III, IV, V, the electron transfer flavoprotein (ETF) complex, ISC complex, and mitochondrial ribosome assembly (21, 22). The acylation of ACP is required for assembly of at least some of these complexes and in the interaction with some of LYRM proteins (15, 23–25), but the status and type of acylation is largely unexplored. Most studies have focused on octanoate, an eight-carbon saturated fatty acid, likely because it is the precursor of lipoic acid. However, a recent study using an MECR variant incapable of extending the acyl chain past octanoate rescued lipoylation but not mitochondrial respiration, suggesting that mtFASII-derived longer chain fatty acids are critical for mitochondrial function (25). In general, mtFASII disruption does not change the major components of the cellular or mitochondrial lipidome (24, 26, 27), but changes have been found in lysophospholipids and ceramides although not always in the same direction (26–28). In addition, MECR binds to the PPAR family of transcription factors activating transcription, suggesting a role for mtFASII in nuclear to mitochondrial communication (29–31). The molecular mechanism by which defective mtFASII leads to mitochondrial dysfunction is an active area of investigation.

Herein, we describe the creation of a mouse model of MEPAN using CRISPR-Cas9 editing resulting in compound heterozygous mutations similar to those identified in most MEPAN individuals. These mice recapitulate the major hallmarks of MEPAN, including a movement disorder and optic neuropathy. Mitochondrial respiration is diminished in the cerebellum of the mutants, but not the cortex, and lipoylation of proteins is reduced in both regions of the brain, but not the retina. Using proteomics and native gel analysis of MEPAN cerebella, we show loss of components of large mitochondrial complexes, and altered formation of supercomplexes of the respiratory chain, delineating a role for acylation by mtFASII in the modulation of these complexes and the downstream effects on movement and balance.

## Results

### CRISPR-Cas9-Targeted Mutations in *Mecr* Yield Viable Pups with Compound Heterozygous Mutations.

**Fig. 1.**
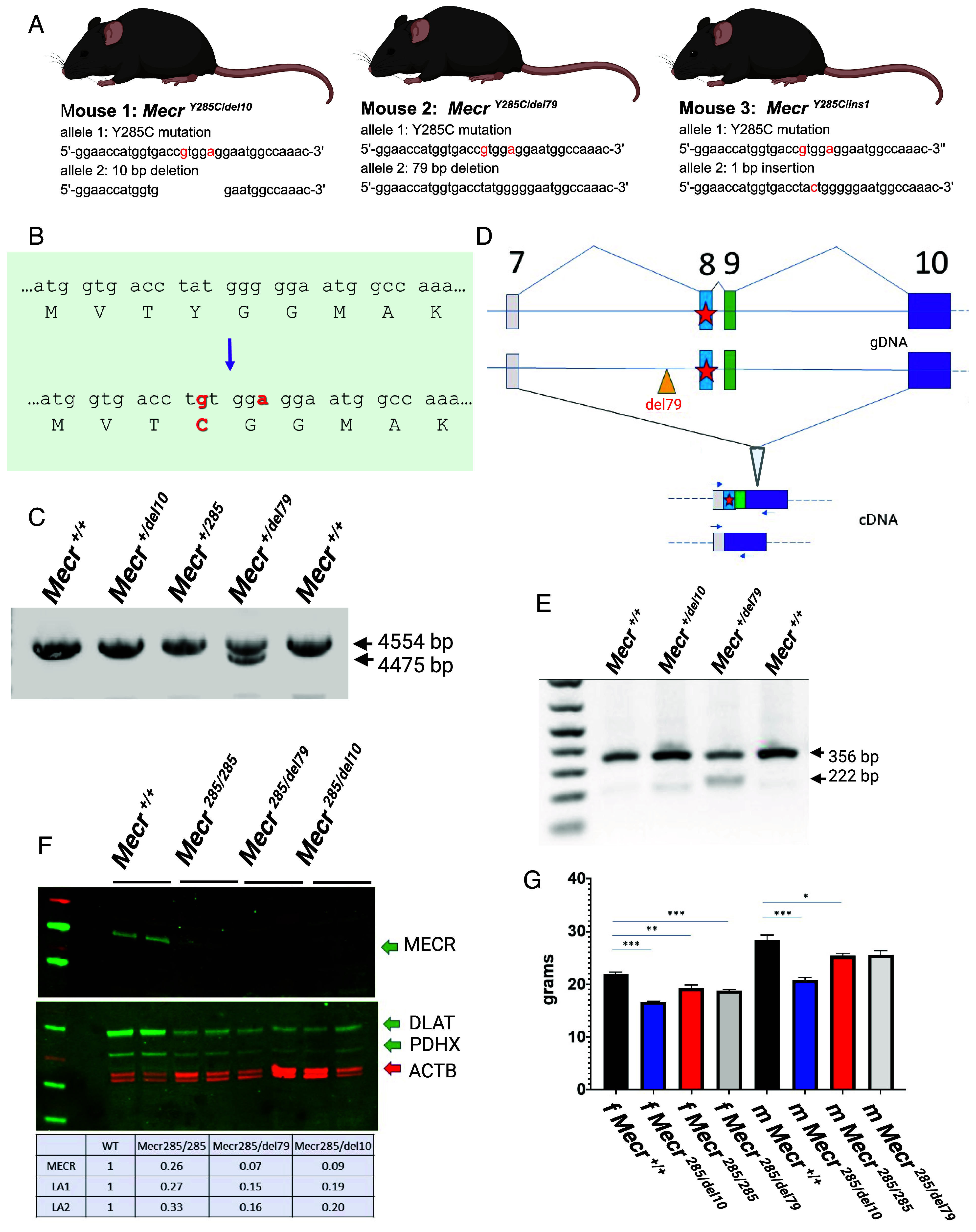
Creation of a mouse model of MEPAN using CRISR-Cas9 genomic editing. (*A*) Sequence of the three mice that were born after CRISPR-Cas9 editing. Mouse 1 had the T285C mutation on one allele and 10 bp deletion on the other allele. Mouse 2 had the T285C mutation on both alleles and an additional 79 bp deletion on one allele. Mouse 3 had the T285C mutation on one allele and a one bp insertion on the other allele. (*B*) The design of the c.854A>G (p.Tyr285Cys) point mutation created by CRISPR-Cas9 editing. The first nucleotide change (*A*–*G* in red) changes MECR tyrosine 285 to a cysteine, a change that is pathogenic in humans. The second mutation (*G*–*A*) is required to create the PAM site for CRISPR-Cas9 editing and is silent. (*C*–*E*, and *G*) Mouse 2 has the T285C point mutation on both alleles (red star in exon 8) and a 79 bp deletion (del79) between exons 7 and 8 on the second allele. (*D*) PCR amplification of genomic DNA from mouse 2 demonstrates the 79 bp deletion. (*E*) Diagram showing alternative splicing of *Mecr* mRNA due to the 79 bp deletion upstream of exon 8 in mouse 2. Alternative splicing of exon 7 to exon 10 in the *Mecr* mRNA eliminates exons 8 and 9, creating a frameshift in the *Mecr* open reading frame. (*F*) MECR protein (*Top* panel) and lipoylation of proteins (*Middle* panel) are reduced in the brain of all *Mecr* mutant mice as determined by SDS-PAGE immunoblotting. Quantitation of bands normalized to actin in shown for all mutants (*Lower* panel). (*G*) All *Mecr* mutant mice have lower body mass at 3 mo of age (*n =* 13–17 per group). (*G*) Amplification of reverse-transcribed RNA from mouse 2 shows del79 results in a smaller cDNA band missing exons 8 and 9.

To create a mouse model of MEPAN disease, we designed guide RNAs to mutate the three *Mecr* codons commonly altered in MEPAN patients: c.695G>A (p.Gly232Glu), c.772C>T (p.Arg258Trp), and c.854A>G (p.Tyr285Cys) (2) using CRISPR-Cas methodology. While over a hundred electroporated zygotes were introduced into pseudopregnant mice for each of the three mutations, only three live pups were born, and all three had been electroporated with the c.854A>G (p.Tyr285Cys) specific guide (Fig. 1 *A* and *B*). Molecular characterization of the *Mecr* locus in these three mice revealed that they each had a different mutant composition. The first mouse had the c.854A>G on one allele of *Mecr* and a 10 bp deletion on the other (Fig. 1*A*). The deletion encompasses the targeted nucleotides, and we assume this is an artifact created by the CRISPR- Cas9 procedure. The 10 bp deletion results in a frameshift of the MECR open reading frame, an addition of 6 nonsense amino acids, and loss of the C-terminal 88 amino acids. The genotype of this mouse was designated *Mecr^285/del10^*. The second mouse had the c.854A>G mutation on both alleles of *Mecr*, but one allele also had a 79 bp deletion 20 bp proximal to the targeted nucleotides in the intron before exon 8 of *Mecr* (Fig. 1 *A* and *C* *Middle* panel). To determine the effect of this intronic deletion, we performed rtPCR on liver RNA from mice with the deletion. In addition to the wild-type *Mecr* cDNA band, a smaller product missing exons 8 and 9 was also amplified from mice with the 79 bp deletion (Fig. 1*E*). Therefore, the 79 bp deletion results in alternative splicing eliminating exons 8 and 9 which code for amino acids 278-324 of *Mecr*, and a frameshift causing an immediate stop mutation in exon 10 (Fig. 1 *D* and *E*). The genotype of this mouse was designated *Mecr ^285/del79^*. The third mouse had the targeted c.854A>G mutation on one chromosome, and a one-base-pair insertion on the other (Fig. 1 *A* *Right* panel). This insertion results in a frameshift, an early stop codon, and truncation of MECR protein at aa 283 with an addition of 18 nonsense amino acids. This mouse died at 4 months of age without giving birth to any progeny.

Crossing mice heterozygous for the c.854A>G mutation resulted in *Mecr^285/285^* in the appropriate 1:4 ratio, demonstrating that homozygous point mutation mice are viable even though they were not obtained by CRISPR-Cas9 methods. The phenotypes of the three viable mutant combinations, *Mecr^285/285^*, *Mecr^285/del10^*, and *Mecr^285/del79^* were evaluated in further experiments. The mutants weighed significantly less than control littermates at 3 to 4 mo of age (Fig. 1*G*). Mutant mice *Mecr^285/285^* and *Mecr^285/del79^* have a normal lifespan while *Mecr ^285/del10^* die suddenly between 3 and 6 mo of age.

### *Mecr* Mutations Destabilize MECR Protein in the Brain.

To ascertain the effect of these mutations on MECR stability, western immunoblots were performed on total protein from the *Mecr* mutant brain. The Tyr285Cys change in MECR results in a 75% reduction in MECR signal in the brain of *Mecr ^285/285^ mice.* The compound heterozygous mutants (*Mecr^285/del10^* and *Mecr^285/del79^*) had half the MECR signal of the homozygous *Mecr ^285/285^* mutants (Fig. 1*F*), suggesting nonsense-mediated mRNA decay or a destabilization of the truncated proteins or both.

### *Mecr* Mutations Have Defective Lipoylation of Protein in the Brain.

An intact mtFASII pathway is required for lipoylation of protein in mitochondria (32–34). To determine whether these *Mecr* mutations result in reduced lipoylation, we performed western immunoblots using an antibody to lipoylated proteins. Two bands corresponding to lipoylated subunits DLAT and PDHX of pyruvate dehydrogenase were decreased commensurate with MECR protein levels (Fig. 1*F*), confirming that mutation of *Mecr* has resulted in compromised protein lipoylation.

#### Localization of MECR in the mouse brain.

**Fig. 2.**
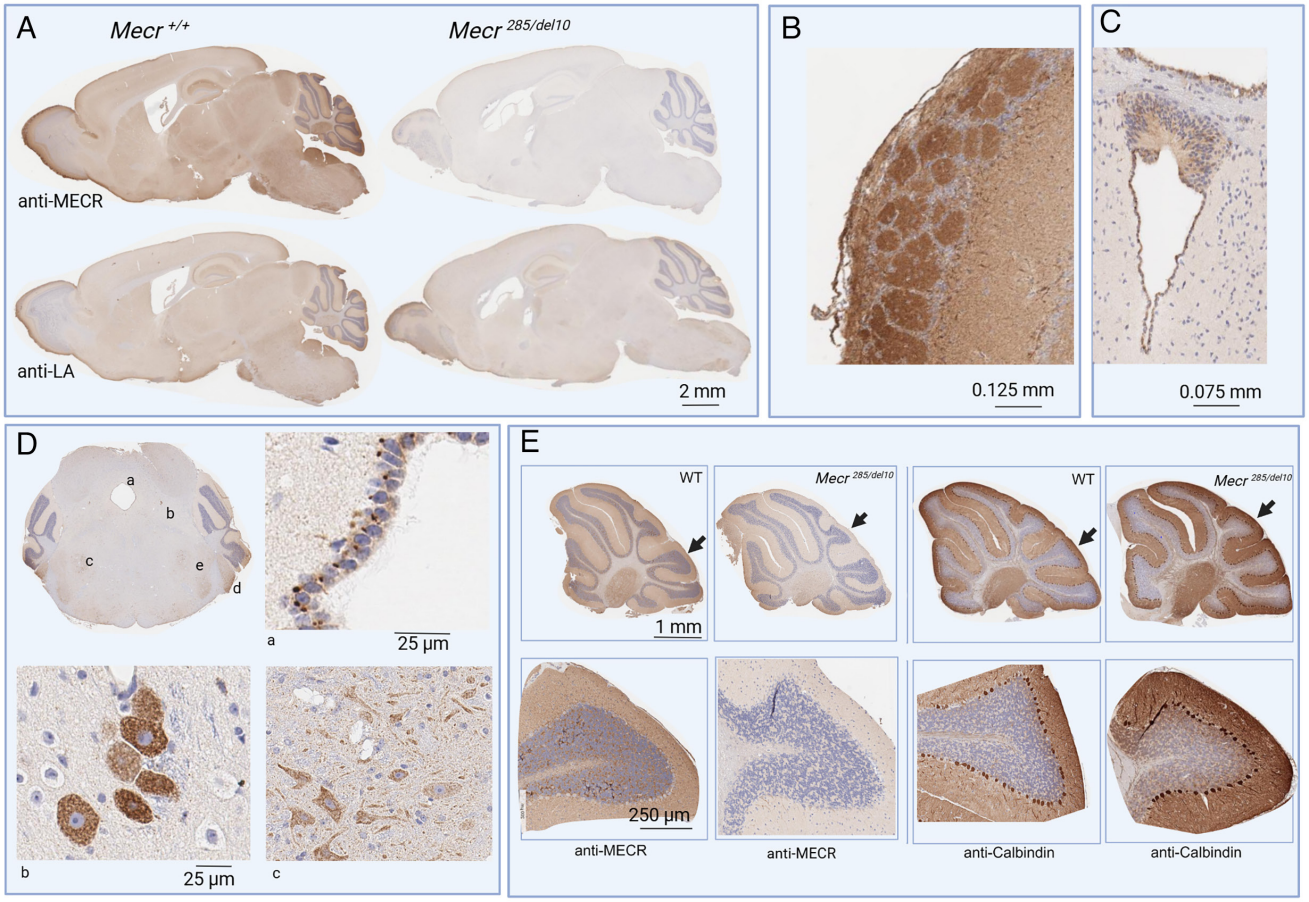
Localization of MECR and lipoylated proteins in the WT and *Mecr ^285/del10^* mouse brain. (*A*) Sagittal sections of whole brain from WT (*Left* side) and *Mecr^285/del10^* (*Right* side) mice were stained with an antibody to MECR (*Top* half) or to lipoylated proteins (*Bottom* half). (*B*) Higher magnification of the olfactory bulb stained with MECR antibody in a WT mouse. (*C*) Higher magnification of tanycytes stained with an MECR antibody in a WT mouse. (*D*) Coronal section of WT brain through the central aqueduct and midbrain/hindbrain. Close-ups of stained regions are a) lining of the central aqueduct, b) midbrain trigeminal nucleus, and c) motor nucleus of the trigeminal nerve. Also labeled are d) the ventral cochlear nucleus and e) the principal sensory nucleus of the trigeminal nerve. E) Sagittal sections of the cerebellum of WT (first and third columns) and *Mecr^285/del10^* (second and fourth columns) with corresponding close-up of one lobe stained with an MECR antibody (*Left* two columns) or a calbindin antibody (*Right* two columns) as a marker of Purkinje cells. (for whole brain, *n =* 4–7 animals for each genotype, for the cerebellum *n =* 4–9 animals for each genotype).

Immunohistochemistry confirmed that MECR and lipoylated proteins are decreased in the brains of the *Mecr^285/del10^* mutant mice (Fig. 2*A*). MECR is most strongly staining the olfactory bulb, the somatosensory region of the cortex, the cerebellum, and the ventral cochlear nucleus. Magnification of the olfactory bulb shows that MECR stains most strongly in the outer layer containing the glomeruli, the globular structures of neuropil that act as the first synapse in the olfactory pathway (Fig. 2*B*). A closer examination reveals MECR localization in specific structures including the periventricular areas of the brain and the choroid plexus in the ventricles (*SI Appendix*, Fig. S1). MECR staining is also strong in the lining of the cerebral aqueduct (Fig. 2 *D, a*). These cells contain large darkly stained mitochondria that are just under 2 μm in width and half the width of the nuclei. Each large mitochondrion is pressed up against a nucleus in a one-to-one ratio on the basal side of the cell. Also staining strongly are tanycytes close to the third ventricle (Fig. 2*C*, coronal section), a group of specialized cells regulating energy balance through regulating feeding and metabolism.

In the posterior coronal sections of the brain (Fig. 2*D*), MECR-specific staining can be seen in several nuclei: the motor nucleus of the trigeminal nerve (Fig. 2 *D, b*), the midbrain trigeminal nucleus (Fig. 2 *D, c*), the primary sensory nucleus of the trigeminal nerve (Fig. 2*D* area e), and the ventral cochlear nucleus (Fig. 2*D* area d). The trigeminal nerve is the cranial nerve that contains three sensory nuclei innervating the skin, mucous membranes, and sinuses of the face, and the motor nucleus innervates the jaw allowing for mastication.

No areas of change between the MEPAN mice and control brain could be seen on examination of H&E-stained sections of the whole brain, either microscopically (areas of necrosis) or macroscopically (size of brain regions).

### MECR Is Not Localized to All Purkinje Cells, and *Mecr* Mutants Do Not Lose Purkinje Cells.

Histological examination of the cerebellum of MEPAN mice showed no major gross structural differences when compared to wild-type mice. MECR staining in the cerebellum is darkest around the deep nuclear layer, less strong but present in the granular layer, and lighter in the molecular layer (Fig. 2*E* and *SI Appendix*, Fig. S1). A limited number of Purkinje cells are stained by MECR antibody (less than 10%) and these cells are within the II, III, and IV/V lobes of the cerebellum specifically, which are associated with inhibition of unnecessary motor movements. To determine whether the Purkinje cells are still present in the *Mecr^285/del10^* mice, Purkinje cell-specific calbindin antibodies were used for immunohistochemistry. Staining of MECR is reduced in the *Mecr ^285/del10^* mice (Fig. 2*E* second column), but Purkinje cells are still present at normal number and distribution (Fig. 2 *E* *Bottom Right*).

### *Mecr* Mutants Have Signs of Retinal Disease That Are Not Caused by Defects in Lipoylation.

MEPAN syndrome patients have optic atrophy, diffuse retinal nerve fiber layer (RNFL) thinning and severe ganglion cell thinning (2). To better understand the manifestation of these phenotypes, we examined MECR localization in the WT and *Mecr ^285/del10^* mouse eye. On gross examination, MECR staining is localized to the retina and cornea of the eye (*SI Appendix*, Fig. S2*A*). In the cornea, MECR is in the superficial stratified layers of the cornea (*SI Appendix*, Fig. S2*B*). In the retina, MECR is most strongly expressed in the ellipsoid zone of the photoreceptor cells where the mitochondria are concentrated, and in the retinal nerve fiber layer (*SI Appendix*, Fig. S2*C*). In addition, magnification of the region between the inner and outer nuclear layers reveals large single mitochondria (*SI Appendix*, Fig. S2*D*) like those seen in the ependymal cells of the brain. These mitochondria are more prevalent on the outer nuclear layer side of the outer plexiform layer, suggesting that they belong to the synapse region of the photoreceptor cells. These large mitochondria are also stained by antibody to lipoylated proteins, which are only found in the mitochondria *SI Appendix*, Fig. S2*E*). To determine the extent of loss of lipoylation in the retina, we stained WT and *Mecr^285/del10^* eyes with antilipoylated protein antibody. The staining was similar in appearance to MECR staining, but with additional staining in the inner plexiform layer (IPL). Surprisingly, staining with antibody to lipoylation in the *Mecr^285/del10^* retina was not decreased, although MECR staining was significantly reduced (*SI Appendix*, Fig. S2*C*, *Middle* panel). Therefore, lipoylation of proteins in the eye is not dramatically affected by MECR mutation. While there is no evidence of alternate lipoylation pathways in mammals, the presence of lipoylated protein in the retina in MECR mutants suggests that enough lipoic acid is being made in the retina, or that turnover of lipoylated protein is slower than in the cerebellum. However, the *Mecr ^285/del10^* retina does have indications of retinal injury or disease as indicated by the increased GFAP staining of Müller glia running through the IPL (3–5). This marker of dysfunction displayed in the *Mecr ^285/del10^* retina in the absence of loss of lipoylation suggests that lipoylation is not the main driver of the pathology in the eye in MEPAN.

### *Mecr* Mutant Mice Have a Movement Disorder, Balance Issues, Altered Gait, and Defects in Olfaction.

**Fig. 3.**
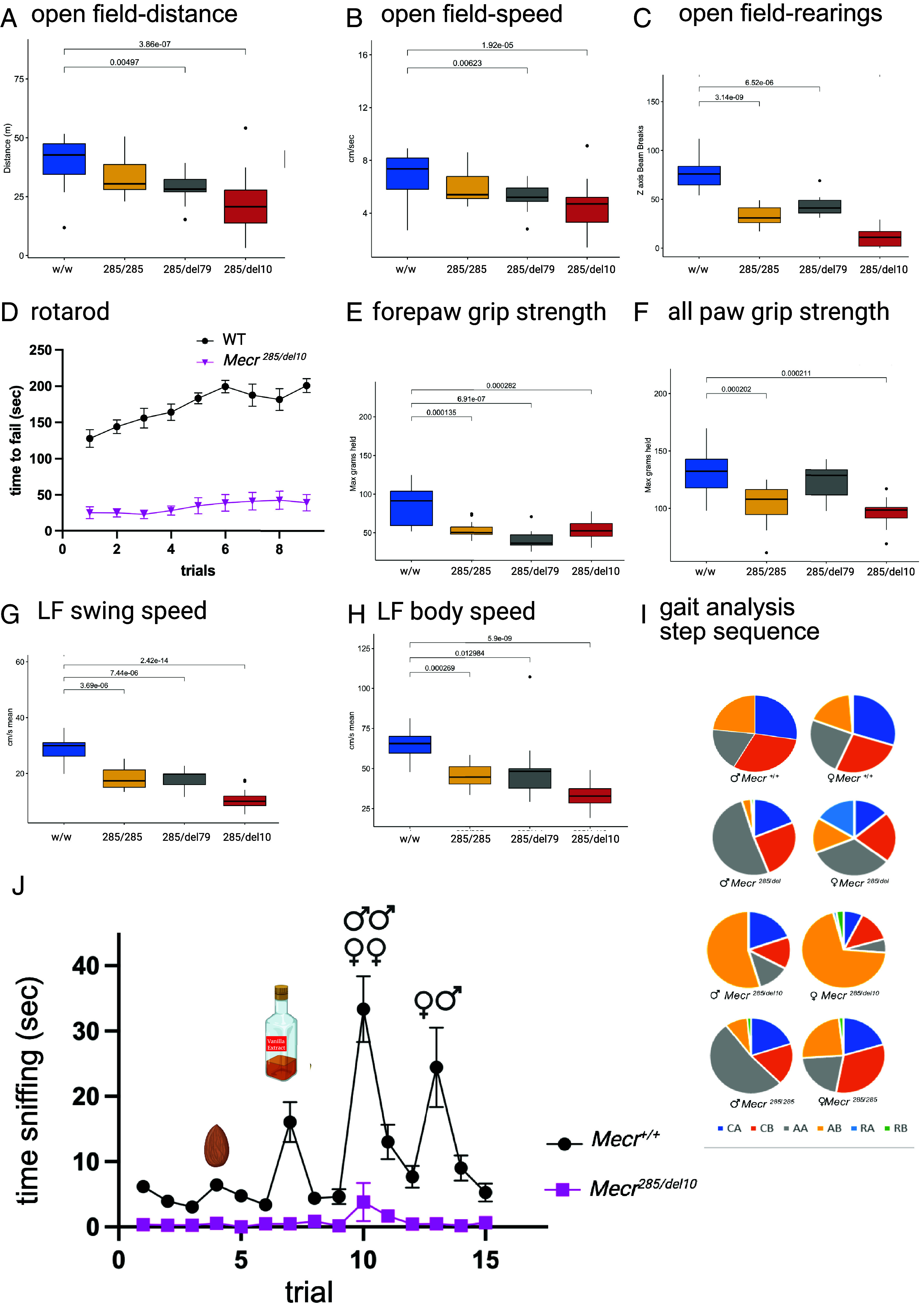
*Mecr ^285/del10^* mice have hallmarks of MEPAN disease, including movement abnormalities and balance deficits, in addition to olfactory dysfunction. (*A*) In the open-field test, the *Mecr* mutant mice show less distance traveled, (*B*) reduced speed of ambulation, and (*C*) fewer rearings (*n =* 9–17 mice per genotype). (*D*) *Mecr^285/del10^* failed significantly more quickly on all of nine trials on the rotarod. WT mice are shown as black circles, and *Mecr^285/del10^* mice are shown as pink triangles. Results shown are the average time to fail with error bars for SE of the mean (n *=* 9–16 mice per genotype). (*E* and *F*) Reduced forepaw and all-paw grip strength in *Mecr* mutant mice (*n =* 9–15 mice per genotype). (G and *H*) are averaged results from analysis of gait testing. All *Mecr* mutants had slower left foot and right foot swing speed as well as decreased body speed (n *=* 9–16 mice per genotype). (*I*) All of the mutants deviated from wild-type mice in step sequence. Gait patterns are AA, RF-RH-LF-LH; AB, LF-RH-RF-LH; CA, RF-LF-RH-LH; CB, LF-RF-LH-RH, where R = right, L = left, F = forepaw, and H = hind paw (*n =* 9–16 mice per genotype). (*J*) Olfactory test. Time spent sniffing in each of fifteen trials was measured. In trials 1–3, the stimulus was water, in trials 4–6, the stimulus was almond scent, in trials 7–9, the stimulus was vanilla scent, in trials 10–12, the stimulus was same sex mouse scent, and in trials 13–15, the stimulus was opposite sex mouse scent (*n =* 6–7 mice per genotype).

MEPAN patients have a movement disorder characterized by ataxic gait, dystonia, and changes in motor coordination (1, 2), and deletion of *Mecr* in Purkinje cells results in a movement disorder (11). To determine whether these patient-similar whole-body mutations also have a movement disorder and to determine whether the created genotypes are the same phenotypically, all three genotypes of *Mecr* mutant mice were subjected to several behavioral tests. Motor coordination and ambulation were tested via the open-field test, rotarod performance, and gait analysis. In the open-field test, *Mecr* mutant mice traveled less distance and had a decreased speed of ambulation (Fig. 3 *A* and *B*) compared to WT mice. Time spent rearing up on their hind legs was also reduced in *Mecr* mutant mice (Fig. 3*C*), indicating muscle weakness, balance issues, or pain. To test balance more directly, an accelerating rotarod test was used which requires the mouse to stay balanced atop a rod that is rotating from 4 to 40 rpm over the course of 300 s. While WT mice could stay atop an accelerating rotarod for an average of 220 s, the mutant mice had significantly decreased latency to fail, with the *Mecr^285/del10^* mice averaging 27 s on the ninth trial (Fig. 3*D*). Since rotarod performance relies on both balance and strength, we measured grip strength in the mutant mice and found that both fore limb and all limb grip strength was significantly decreased (Fig. 3 *E* and *F*), suggesting that balance might not be the only factor in their performance.

We also performed detailed gait analysis for the *Mecr* mutant mice using the Noldus Catwalk system. The mutants had significantly slower body speed (Fig. 3*H*) recapitulating open-field analysis results, and significantly slower bilateral foot swing speed (Fig. 3*G*). Significant differences were also seen in the step sequence between WT and mutant mice (Fig. 3 *E*–*I*). The WT mice use the four common step sequences (CA, CB, AA, and AB) at roughly equal amounts, while the mutants have a shift toward the AA sequence (*Mecr^285/del^* and *Mecr^285/285^*) or the AB sequence (*Mecr^285/del10^*). Alterations in step sequence is described as a gait parameter specific to coordination (35). In summary, *Mecr* mutant mice move more slowly than wild-type mice, have defects in balance, and exhibit altered gait patterns along with some general muscle weakness, similar to MEPAN patients.

Because MECR stains most darkly in the olfactory bulb of the brain (Fig. 2 *A* and *B*) we investigated sense of smell in the mutant mice. The olfaction test involves measuring the response of a mouse to different smells by recording the amount of time it spends sniffing when a new odor (almond, vanilla, same sex mouse, or opposite sex mouse) is introduced. *Mecr^285/del10^* mice showed little to no attention to the changing odors, although they did move about the cage, suggesting a complete loss of olfaction in these animals (Fig. 3*J*).

### Reduced Respiration in the Cerebellum but Not the Cortex of *Mecr* Mutant Mice.

**Fig. 4.**
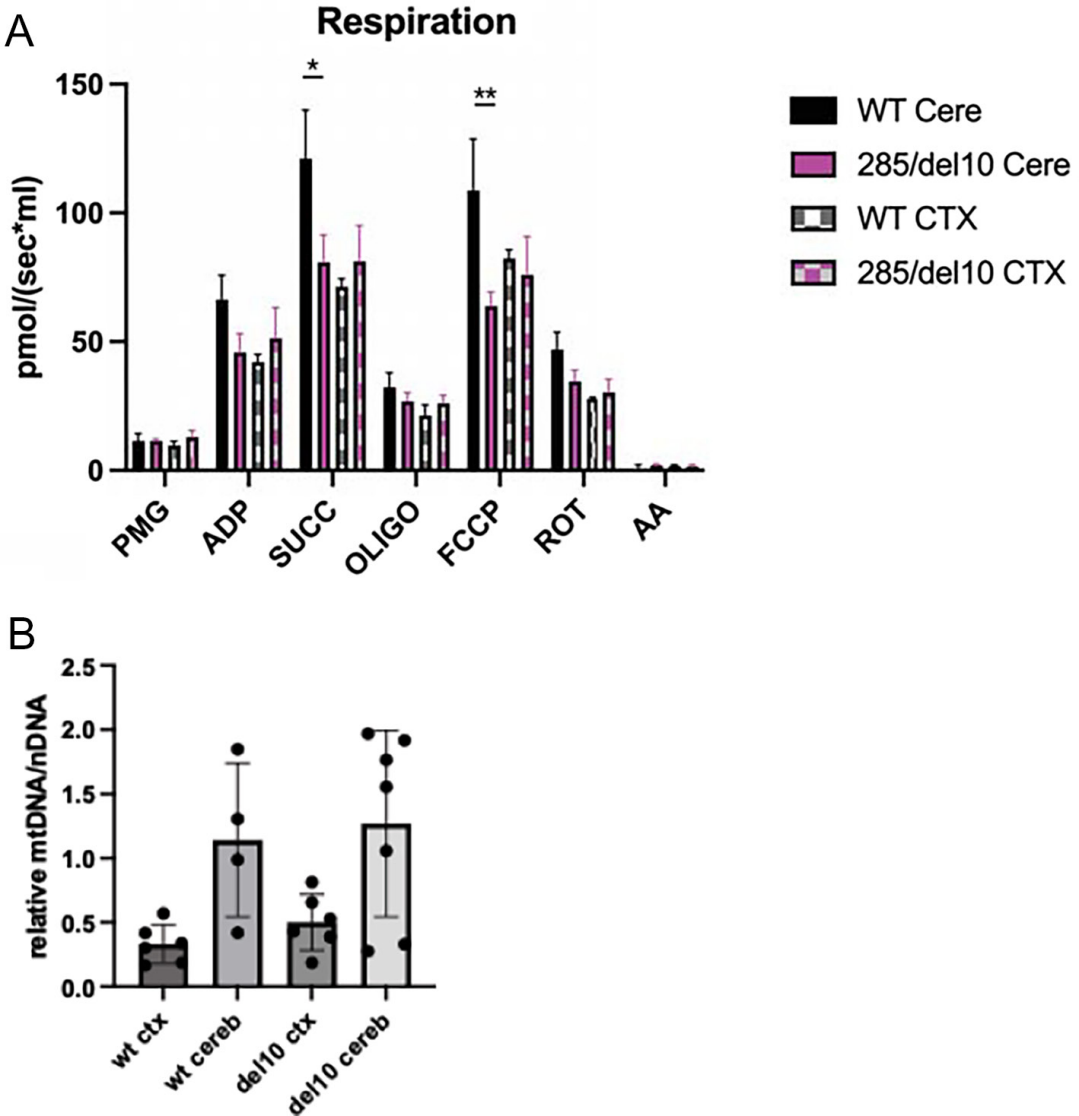
*Mecr* mutant mice have reduced respiration in the cerebellum, but not the cortex, compared to wild-type mice. (*A*) Respiration in the cortex and cerebellum of WT vs *Mecr^285/del10^* mice. PMG = complex I-linked leak respiration (substrates pyruvate, malate, and glutamate); ADP = ADP stimulated; SUCC= OXPHOS capacity with PMG and succinate; OLIGO = leak respiration with oligomycin; FCCP = uncoupled mitochondria; Rot = rotenone inhibition of complex I demonstrating complex II-linked respiration AA= antimycin A *(n =* 4–5 mice per genotype). (*B*) Ratio of mitochondrial DNA (mtDNA) to nuclear DNA (nDNA) in the cortex (ctx) and cerebellum (cereb) of wild-type (wt) and *Mecr^285/del10^* (del10) mice as measured by quantitative real-time PCR normalized to ratio in the wild-type cerebellum. * = *P* < 0.05; ** = *P* < 0.005.

To determine whether the balance and movement disorder of the MEPAN mouse is due to defects in mitochondrial respiration in the cerebellum, we performed high-resolution respirometry. The *Mecr^285/del10^* mice showed a global reduction in mitochondrial respiration in the cerebellum relative to WT mice (Fig. 4*A*). This included a significant decrease in complex I and II–linked respiration (substrates pyruvate, malate, glutamate, and succinate, “SUCC”), and maximal respiration (“FCCP”). A similar decrease in global respiration was not seen in the cortex of the *Mecr^285/del10^* mice, and respiration was unchanged across all tested parameters (Fig. 4*A*). *Mecr^285/del10^* have the same amount of mtDNA in both the cortex and cerebellum as WT mice (Fig. 4*B*), hence the change in cerebellar respiration in the mutant mice is independent of mtDNA content.

### Proteomic Analysis of *Mecr* Mutant Cerebella Identifies Defects in the Iron–Sulfur Cluster Assembly Complex.

**Fig. 5.**
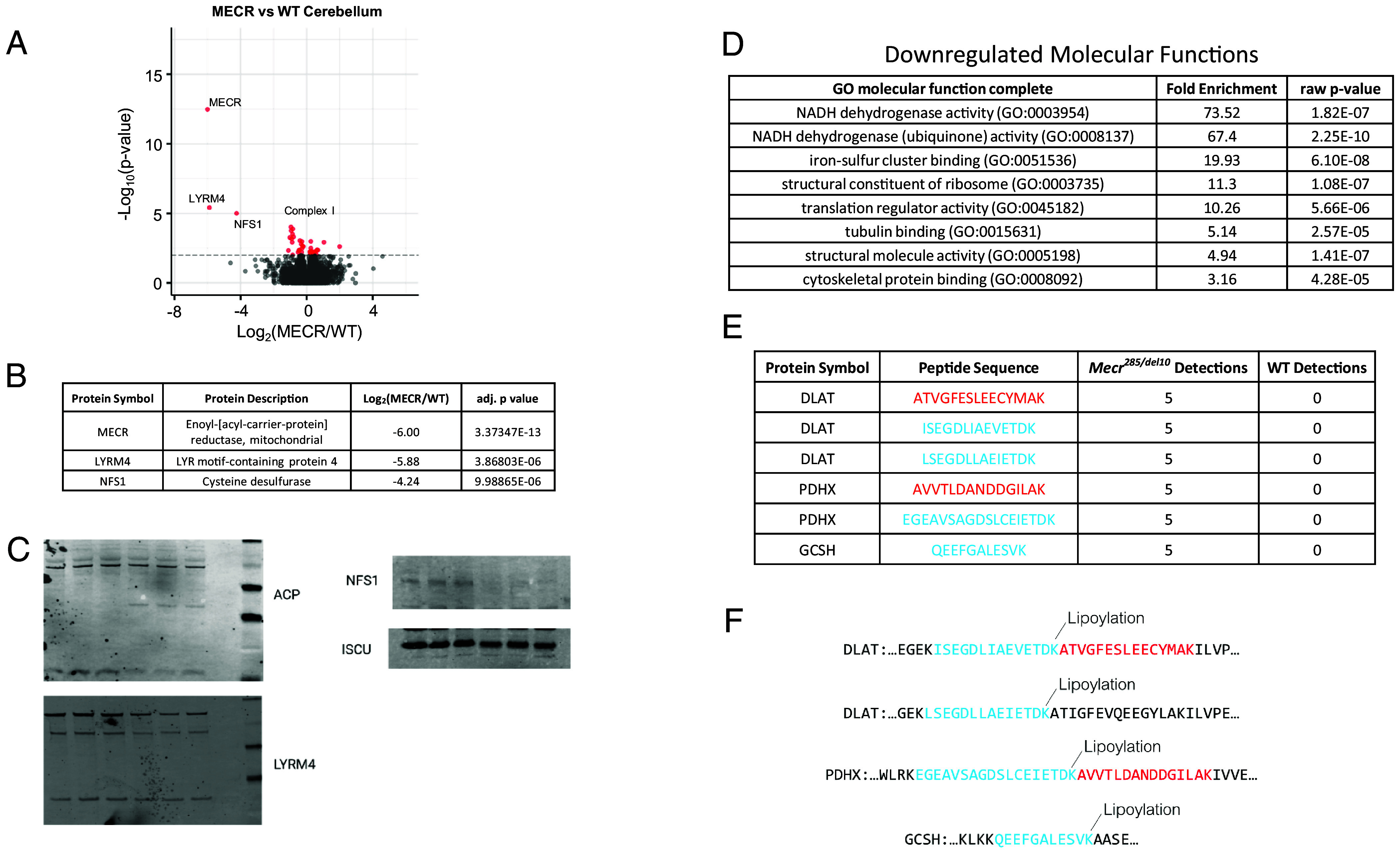
Proteomics analysis shows changes in several groups of proteins in the *Mecr^285/del10^* mutant cerebellum. (*A*) Volcano plot of comparison of proteins in the WT and *Mecr^285/del10^*cerebellum. Components of complex I of the electron transport chain are circled. (*B*) Top three downregulated proteins in the *Mecr^285/del10^*cerebellum are MECR followed by LYRM4 and NFS, components of the iron–sulfur cluster assembly complex. (*C*) Representative images of western analysis of ISC assembly complex LYRM4, NFS, and ACP (*n =* 3 mice per genotype per blot and were repeated at least twice). (*D*) List of molecular functions significantly downregulated in the *Mecr^285/del10^*cerebellum, their fold enrichment, and *P* value as determined by pathway analysis. (*E* and *F*) Defective lipoylation of proteins can be identified in mass spectrometry data by unmasking of trypsin sites.

**Fig. 6.**
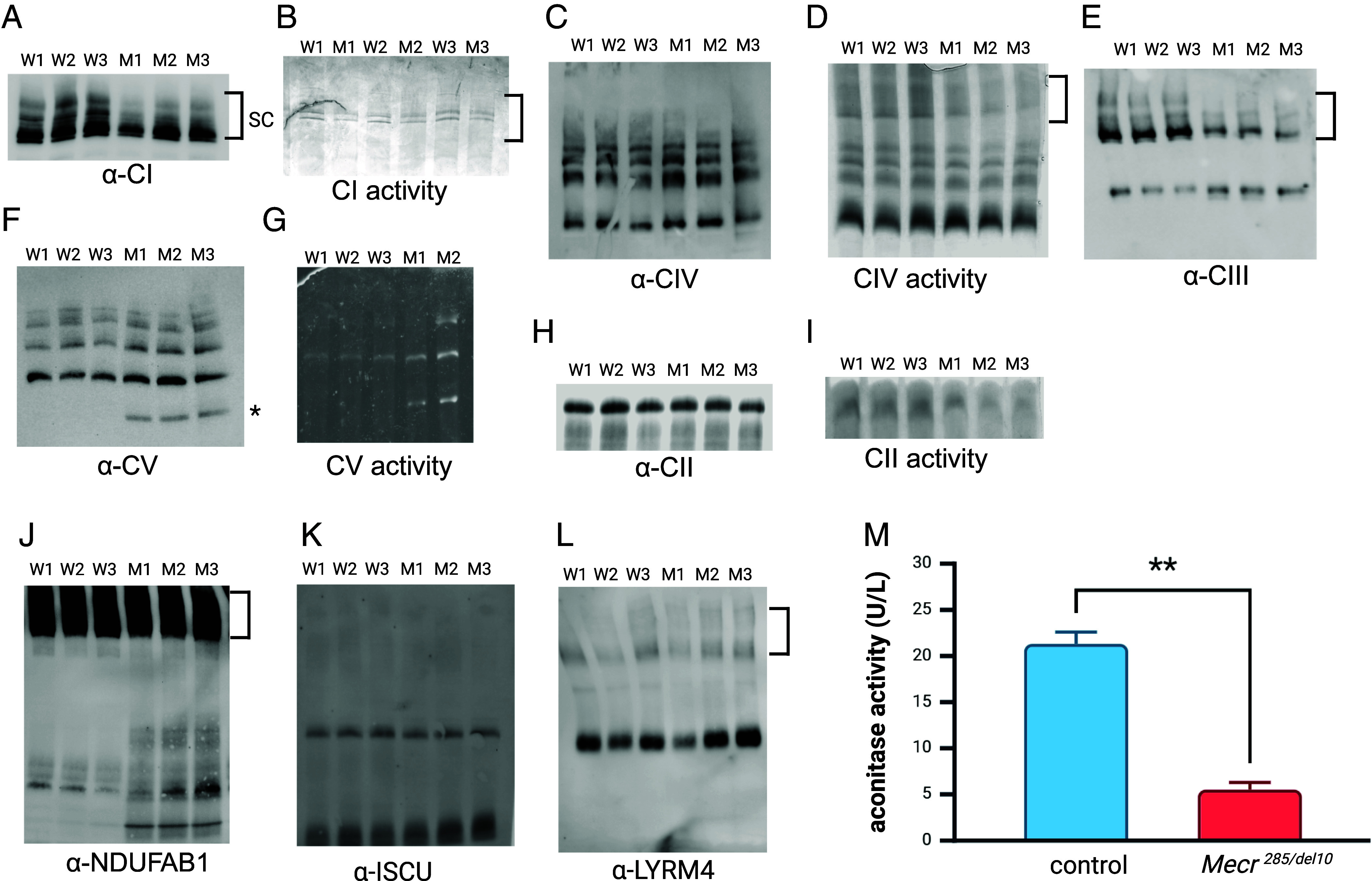
Mitochondrial respiratory complex formation and activity are altered in the *Mecr^285/del10^* (M1-3) cerebellum. Representative immunoblots (*A*, *C*, *E*, *F*, *H*, *I*, *K*, and *L*) and in-gel activity assays (*B*, *D*, *G*, and *I*) of mitochondrial proteins run on native PAGE gels. Gels were transferred to PVDF and probed with antibody to (*A*) complex I, (*C*) complex IV, (*E*) complex III,) (*F*) complex V, (*H*) complex II. (*J*) ACP (NDUFAB1), and components of the ISC assembly complex (*K*) α-ISCU and (*L*) α-LYRM4. (*M*) Aconitase activity in the cerebellum of WT and *Mecr^285/del10^* mice (*n =* 3 mice per genotype and repeated at least twice). W = wild type; M = *Mecr^285/del10^*.

To better understand the mitochondrial dysfunction in the cerebellum of *Mecr* mutant mice, we performed LC–MS/MS-based proteomics using data-independent acquisition. The top three most significantly downregulated proteins are MECR, LYRM4, and NFS1 (Fig. 5 *A*–*C*). LYRM4 and NFS1 are essential components of the mitochondrial iron–sulfur (Fe/S) cluster (ISC) assembly complex along with ISCU which is also downregulated in the *Mecr^285/del10^* cerebellum. The ISC assembly complex creates Fe/S clusters essential for the catalytic activity of several enzymes, including complex I, II, and III of the mitochondrial electron transport chain, lipoic acid synthase, and aconitase. The ISC assembly complex is the prototypical LYRM/ACP interaction complex (16, 36), where LYRM4 (ISD11) interacts with ACP through its acylated pantothenate group (18), followed by acyl chain “flipping” from the ACP to a hydrophobic domain in LYRM4 (18, 37). We hypothesized that a loss of acylation of ACP due to mtFASII dysfunction in *Mecr* mutants results in the destabilization of the ISC complex assembly apparatus. Indeed, immunoblots with ACP antibodies demonstrated a decrease in the acylated form of ACP, and a concomitant increase in the 28 kD form of ACP which is dimerized through its unacylated pantothenate group (36, 38) (Fig. 5*C*). Loss of iron–sulfur clusters from destabilization of ISC complex assembly would be expected to result in decreased activity of FeS cluster-dependent enzymes like aconitase. While mitochondrial aconitase protein levels are not significantly different in *Mecr* mutants, its activity was found to be 50% of control (Fig. 6*M*), consistent with loss of FeS centers.

### Subunits of Complex I Are Reduced in the *Mecr^285/del10^* Cerebellum.

The next seven proteins with the most significant altered levels in the mutant cerebellum were all components of complex I (NADH dehydrogenase) of the electron transport chain. Of the 47 complex I structural and accessory proteins identified by mass spectrometry, 12 were significantly lower in the cerebellum of the *Mecr^285/del10^* mutant (*SI Appendix*, Fig. S3) and one protein, assembly factor (NDUFAF3), was upregulated. These 12 down-regulated proteins are predominantly in the matrix arm (12/13) of complex I (39) and their downregulation was confirmed by western immunoblotting (*SI Appendix*, Fig. S2). The crystal structure of complex I contains ACP at two sites interacting with two LYRM proteins, LYRM3 (NDUFB9) and LYRM6 (NDUFA6) which are both a part of the ACP interactome (40). NDUFA6 is downregulated in the mutant cerebellum, while NDUFB9 is unchanged. The upregulated assembly factor, NDUFAF3, is not considered a LYRM protein but contains a LYR motif toward its N-terminus in humans and an LFR motif in mice. Interestingly, knock out of another LYRM protein, LYRM2, causes loss of a similar profile of N-module proteins in HEK293 cells (40). LYRM2 interacts with ACP and complex I and upregulates OXPHOS in colon cancer through integration of the N-module for efficient assembly of complex I (41), but it is not represented in our cerebellar proteomic data. Loss of acylation of ACP in *Mecr* mutants could result in altered complex I assembly through any or all these LYRM proteins in the cerebellum. Alternatively, the destabilization of complex I in *Mecr* mutants may be a downstream effect of disruption of the ISC assembly complex, since complex I contains eight of the 12 Fe–S clusters found within the ETC complexes, and all are located within the peripheral matrix arm (42).

Because of the predominance of complex I subunits in the list of significantly changed proteins, we examined whether the proteins of the other complexes of OXPHOS were altered in the LC-MS/MS dataset. Of 48 structural subunits and assembly factors of complexes II through V, none were significantly altered.

### LC–MS/MS Analysis Identifies Loss of Protein Lipoylation in the *Mecr*^285/del10^ Cerebellum.

LC-MS/MS analysis also allowed us to infer loss of lipoylation of several proteins due to peptide dropouts (Fig. 5 *E* and *F*). Lipoylation of lysine, which blocks tryptic digestion, is canonically present on K104 of GCSH. However, the tryptic peptide 94-QEEFGALESVK-104 of GCSH was found only in the mutant mice, suggesting that there is incomplete lipoylation in the *Mecr* cerebellum (Fig. 5 *E* and *F*). Canonical lipoylation sites were also unblocked in the mutant mice in the dihydrolipoyllysine-residue acetyltransferase component of pyruvate dehydrogenase complex at K131 and K258, and the protein X component of pyruvate dehydrogenase at K97. The defect in lipoylation in *Mecr* mutants has been attributed to the requirement for mtFASII-derived octanoate to make lipoic acid. Since lipoic acid synthase also requires an iron–sulfur center to synthesize lipoic acid, the defect in ISC assembly machinery in *Mecr* mutants could also contribute to the loss of lipoic acid.

Gene ontology (GO) analysis of the proteins significantly changed in the cerebellum identified several molecular functions that are downregulated in the *Mecr^285/del10^* mutants, including the aforementioned NADH dehydrogenase (ubiquinone) activity and the 2 iron–2 sulfur cluster function (Fig. 5*D*). Also downregulated were the structural constituent of the ribosome (GO: 0003735) and the translation regulator activity function (GO:003734). Of note, similarities were identified in network analysis of proteins interacting with ACP (40), in which ribosome assembly is modulated by interaction of ACP with the LYRM protein AltMiD5, and complex I assembly by interaction of ACP with LYRM2 in HEK293 cells. Two of the remaining downregulated molecular functions (tubulin binding and cytoskeletal protein binding) are related to the cytoskeleton but have no known association with ACP or LYRM proteins.

### Altered ETC Complex/Supercomplex Formation in the *Mecr^285/del10^* Cerebellum.

Complex I (NADH dehydrogenase) is a large protein complex that exists in larger supercomplex forms with complexes III (ubiquinone cytochrome C oxidoreductase) and IV (cytochrome oxidase). To determine whether *Mecr* dysfunction is associated with changes in complex/supercomplex formation, western blotting of nondenaturing polyacrylamide electrophoresis (native PAGE) was performed. Antibodies to complex I (α-NDUFV2) show that a great preponderance of complex I is detected in four supercomplex forms in the wild-type cerebellum and that these supercomplexes are decreased in the *Mecr* mutant cerebellum (Fig. 6 *A*–*C* and *E*). Antibodies to complex III (UQCRC1) and in gel activity assays of complex IV confirm that supercomplex amount and activity is down in the *Mecr* mutant cerebellum (Fig. 6 *C*–*E*). In addition, independent dimers of complex III are increased in the Mecr mutant cerebellum, presumably because they are redistributed from supercomplexes (Fig. 6*E*). Complex II (succinate dehydrogenase, SDH) does not participate in supercomplexes, and the amount of the complex as detected by α-SDHA antibody is not changed in *Mecr* mutants (Fig. 6*H*) consistent with proteomics results. SDH does rely on FeS clusters for activity, and in gel activity assay of SDH is decreased in the *Mecr* mutant cerebellum, providing evidence of the loss of iron–sulfur clusters due to ISC biogenesis dysfunction (Fig. 6I). Complex V (ATP Synthase) is similarly not a part of supercomplex formation, and α-ATP5A antibody identified bands on native immunoblot corresponding to monomer, dimer, trimer, and tetramer forms of ATP synthase in the mouse cerebellum (Fig. 6*F*). While these multimer forms are not changed in *Mecr^285/del10^* mice, another band is identified that corresponds to the F1 subunit of ATP synthase suggesting that the two modules of ATP synthase have come apart in the *Mecr* mutants (Fig. 6*F*). Disruption of a LYRM/ACP protein complex specific for ATP synthase would explain the structural changes in ATP synthase in the *Mecr* mutant cerebellum. This phenotype is similar to that of LYRM protein FMC1 deficiency in *Saccharomyces cerevisiae,* where the F1 subunit of ATP synthase does not assemble onto the F0 subunit under high temperature stress (43), and to knock down of LYRM protein altMID51 in HEK293T cells (40). In gel assay of ATP synthase activity showed that it is not decreased in the *Mecr* mutant cerebellum but may in fact be increased (Fig. 6*G*).

Since ACP is found in crystal structures of complex I and the ISC complex, we used antibodies to ACP to probe native gels and found that respiratory supercomplexes were the most strongly stained with α-ACP in both control and mutant cerebellum (Fig. 6*J*). In addition, α-ACP stains a complex in the *Mecr* mutant cerebellum that is the size of the ISC assembly complex as compared to native gel staining with α-ISCU and α-LYRM4 Fig. 6 *K* and *L*). In yeast, the ISC assembly complex is associated with respiratory supercomplexes through interactions with complex III and IV (44). Interestingly, α-LYRM4 identifies supercomplexes in the mouse cerebellum in our study (Fig. 6*L*), suggesting the same is true in mammalian systems. In the *Mecr* mutant cerebellum, α-ACP also stains non-supercomplex-associated ISC assembly machinery suggesting that *Mecr* mutants are not efficiently integrating ISC machinery into supercomplexes. (Fig. 6 *J* and *L*). There are other unidentified bands staining more intensely with ACP antibody in the *Mecr* mutants than in the wild-type cerebellum. In HEK293 cells, the interaction network of ACP contains proteins from all five OXPHOS complexes, in addition to the iron–sulfur cluster assembly complex and the mitochondrial ribosome (40), and all of these proteins are candidates for the unidentified complexes, in addition to the electron transfer flavoprotein complex which interacts with LYRM5 and contains an iron–sulfur center (45, 46). Investigation into the components of these bands interacting with ACP requires further investigation.

## Discussion

We have created a mouse model of MEPAN syndrome with compound heterozygous mutations recapitulating the main hallmarks of the disease. While other mouse models of mtFASII dysfunction exist, including an inducible MCAT deletion model (33) and a Purkinje cell-specific *Mecr* knock-out model (11), the mutations described herein provide several advances in mirroring the patient phenotype, including the evaluation of the effect of these mutations on other tissues, and in all regions of the brain. We show that MECR expression is not limited to the Purkinje cells or the cerebellum but has region-specific expression throughout the brain and the eye. While we focused mechanistic studies on the cerebellum, individuals affected by MEPAN are characterized by T2 signal abnormalities in the basal ganglia. This is similar to Leigh syndrome, a progressive neurodegenerative disorder of early childhood secondary to mitochondrial dysfunction which is characterized by T2 signal abnormalities in the basal ganglia and brain stem. In the mouse model of Leigh syndrome, T2 signal hyperintensity has been linked to gliosis (Quintana et al 2012), and microglia and peripheral macrophages play a key role in the pathophysiology of Leigh syndrome. T2-weighted MRI studies linked to histological studies of gliosis in the MEPAN mice might help to better understand the mechanistic basis of T2 hyperintensities in mitochondrial disease.

The intense staining of MECR in the glomeruli of the olfactory bulb led us to investigate olfaction in these mice, and find that it is dramatically reduced, supporting the hypothesis that MECR protein levels in the brain highlight those regions most reliant on MECR. One individual with MEPAN reports no decrease in olfaction, but a complete survey of all MEPAN individuals is necessary. The presence of large mitochondria stained with MECR antibody in the ependyma and retina suggests a unique role for MECR in these tissues that begs further investigation. The phenotype of the MEPAN mice fits the definition of multiple mitochondrial dysfunction syndrome, in that they have respiration defects that are not tied to one specific complex of OXPHOS. Complexes I, II, III, and IV are all dysfunctional in MEPAN mice due to complex/supercomplex destabilization and/or loss of FeS clusters. This is especially relevant in the retina of the MEPAN mice where retinal dysfunction exists but lipoylation is not decreased, suggesting that supercomplex and FeS cluster formation are the root cause of the retinal phenotype. The data presented in this paper connect the acylation of ACP (by mtFASII) and the interaction with LYRM proteins in the proper functioning of mitochondrial energy production through FeS cluster assembly and respiratory complex/supercomplex formation.

In the *Mecr^285/del10^* cerebellum, the loss of acylation of ACP results in destabilization of the ISC assembly complex and the inability to integrate it into respiratory supercomplexes. The interaction of acyl-ACP and LYRM4 in the assembly and structure of the ISC assembly complex is well studied and serves as a paradigm for role of ACP/LYRM proteins in the assembly of other large protein complexes (15, 18, 47). In the crystal structure of the ISC complex, ACP is on the outside of the complex interacting only through LYRM4 (47), and the interaction of ACP with LYRM4 is proposed to be necessary for the binding and stabilization of NFS in the complex (48). ACP interacts with LYRM4 through the phosphopantetheine group on ACP, and the flipping of the acyl chain from ACP to a hydrophobic domain on LYRM4 is an integral part of the assembly process (15, 21, 37).

The molecular results presented here have a common thread of the role of MECR and mtFASII in the acylation of ACP, its interaction with LYRM proteins, and their role in formation of FeS clusters and large protein complexes in the mitochondria. It is a perhaps overlooked fact that the characterized LYRM proteins all play roles in assembly of complexes that synthesize or contain FeS clusters: complex I, complex II, and complex III of the respiratory chain, the mitochondrial ribosome, the ISC assembly complex, and the electron transfer flavoprotein. The one exception to this rule is noncanonical LYRM protein FMC1 and its role in ATP synthase complex assembly. ATP synthase does not have an ISC, but there is evidence that it plays a role in mitochondrial iron uptake (49, 50). These data and complementary data in the literature favor a model in which the acylation of ACP by mtFASII and its interaction with LYRM proteins have all evolved to protect the cell from the toxic interaction of Fe and O_2_. FeS clusters are ancient structures that can form spontaneously in anaerobic conditions but are labile in the oxygen-rich conditions that evolved on earth. The fact that components of the ISC assembly machinery (including FXN, ISCU, GLXN5, NUBPL, SFXN1, BOLA3, FDX1, NFS1, and HSCB) and mtFASII components (including MECR, MCAT, and OXSM) are protective against high oxygen levels (51) suggests that they have evolved to protect the cell from damaging oxygen radicals that occur when Fe and oxygen meet. This is also validated by the observed therapeutic effect of hypoxia on the mouse model of Friedreichs ataxia (52), which has a mutation in frataxin, a component of the ISC assembly apparatus. Evolutionarily, LYRM proteins do not exist in bacteria, although the complexes that LYRM proteins facilitate do (53). LYRM proteins appear in eukaryotes and are lost when eukaryotes evolve in an anaerobic environment. The broader role of ACP in coordination of mitochondrial energy metabolism and perhaps oxygen toxicity is also witnessed by the cardioprotective effect of ACP overexpression in ischemia–reperfusion injury (54). The *Mecr* mutant mouse model demonstrates the critical role of mtFASII-driven acylation of ACP in the modulation of multiple convergent processes critical to efficient mitochondrial respiration.

The in situ structure of porcine heart supercomplexes has been elucidated with cryoEM, defining the stoichiometry and abundance of the various forms in the heart (55). The four main assemblies consist of complexes I, III, and IV in the ratios I_1_III_2_IV_1_, I_1_III_2_IV_2_, I_2_III_2_IV_2_, and I_2_III_4_IV_2._ A striking feature of the in situ structure of the supercomplexes is that the interstitial space between CI, CIII_2_, and CIV is populated by lipids that mediate the interactions between complexes, and no direct protein–protein interactions between CIII_2_ and CIV were observed (55) raising the possibility that lipids created by mtFASII might play a role in the physical interaction of the supercomplexes. Notably, all the ISC structures published recently were created by coexpressing the human subunits (ISCU, LYRM4, NFS, and frataxin) in *E. coli* and in the presence of *E. coli* ACP and bacterial FASII. It is possible that these conditions could underlie structural differences due to differences in ACP because of altered acyl chain formation and modification in bacteria compared to mitochondria. Very little is known about acyl chain composition in the ACP/LYRM modulation of large protein complexes in mammalian systems, and the *Mecr* mutant mouse provides a model for studying these variables. Fatty acids can differ by carbon length, saturation, and other modifications (hydroxylation, amination, etc), and the acyl chain on ACP could vary by tissue and/or metabolic state. The role of the identity of the acyl chain in ACP/LYRM modulation is an active area of research.

## Materials and Methods

### Animals.

All animals were cared for in accordance with the ethical guidelines of the National Institutes of Health and the Institutional Animal Care and Use Committees of The Children’s Hospital of Philadelphia and University of Pennsylvania. The mice were fed 5LOD diet from PicoLab® Laboratory and were maintained on a 13:11 h light–dark cycle.

### Creation of *Mecr* Mutant Mice with CRISPR-Cas9 Editing.

CRISPR/Cas9-mediated gene editing was performed in zygotes of C57BL/6 miceA guide RNA targeting three genomic regions of interest, Cas9, and single-stranded DNA containing mutant sequence were electroporated into zygotes as described (56, 57). CRISPR reagents were purchased from Integrated DNA Technologies, Inc. The RNA component and donor templates are listed in *SI Appendix*, Table S1

### Accelerating Rotarod.

The Rotarod (IITC San Diego Ca.) is a one-inch diameter, horizontal rod with a coarse surface, programmable to accelerate at different rates. The mouse must adjust the cadence of its stride to remain on the accelerating rod. Three trials per day were performed over three consecutive days with the Rotarod accelerating from four to forty rpm in three hundred seconds. On the first trial, the mouse was habituated to the stationary rod for 2 min before rotation begins. A trial ends when the mouse fails to walk, by either falling from the rod or making a full rotation while gripping the rod.

### Gait Analysis.

The Catwalk XT (Noldus Information Technology, Netherlands) is a sophisticated system used to acquire quantitative gait and paw placement dynamics. After a 30-min habituation to the darkened procedure room, a trial began by placing a mouse at one end of a forty cm enclosed alley. The mouse walks freely toward a goal box containing its home cage. Data were collected over a twenty cm sampling region in the middle of the alley. Footfalls, illuminated from below, were pixelated to collected data on limb motion, stride dynamics, and ambulation patterns. Additionally, the pixelated paw print data provide measures of load, braking, standing, and propulsion during passive ambulation. At least five runs were collected for each mouse then post hoc visual validation of runs provided at least three compliant runs for each mouse for analysis.

### Open-Field Activity.

After a 30-min habituation to the testing room, a 10-min trial began with a mouse placed in the center of an open-field arena (16″ × 16″ × 15″). The arena is fitted with a scaffold of IR emitters and detectors to collect peripheral, center, and vertical (rearing) beam breaks. The Photobeam Activity System (San Diego Instruments) assessed spontaneous locomotion and rearing activity. In addition, all trials were recorded by high-definition camcorders. Digitally recorded trials were processed for automated analysis by ANYmaze software (Stoelting Co, Il) to provide distance and time metrics for speed of ambulation and time in center measures (>2″ from the wall).

### Grip Strength.

Grip strength meters are used routinely to assess strength as part of a general health assessment. After 30 min of habituation to the procedure room, a Grip Strength Meter (IITC Instruments, San Diego, Ca) is used to measure forepaw and hindpaw grip strength on subsequent days. For forepaws, a mouse is lowered by the tail so that it grasps a metal T bar with forepaws only. The mouse is pulled backward slowly by the tail in the horizontal plane to exert a force on the T bar, which is transduced to a meter that records the maximum force (grams) exerted before the release of the bar. Five trials are performed with a 20-min intertrial interval. 24 h after the forepaw trails, hindpaw grip strength is measured. For hindpaws, the T bar is replaced with a chicken wire grid. Similarly to the forepaw test, the mouse is allowed to grasp the grid, now with all four paws then slowly pulled backward so that the maximum force (grams) exerted before the release of the grid is obtained. Five trials are performed with a 20-min intertrial interval.

### Olfactory Habituation–Dishabituation.

To assess dynamic olfactory function, we used a habituation–dishabituation procedure (58). Briefly, a cotton-tipped applicator is suspended from the lid of a single-housed mouse to present the odorants. In order, the odorants used were 1) water (neutral control), 2) almond extract, 3) vanilla extract (McCormick extracts at 1:100 dilution), 4) same sex cage, and 5) opposite sex cage. Social odorants were obtained by wiping the cage floor of mice in the colony room. Each odorant was presented in three consecutive 2-min trials with a 1-min intertrial interval. Habituation to each odorant was determined by the time sniffing the applicators on successive trials of the same odorant. Dishabituation was determined by comparing the sniffing time of the third trial for each odorant to the first trail of the subsequent novel odorant. All trials were performed in dim light and recorded for offline grading by a highly trained person, blind to group designation,

### Immunohistochemistry.

For histology of the cerebellum and eyes, mice were euthanized by cervical dislocation under the Institutional Animal Care and Use Committee (IACUC) of The Children’s Hospital of Philadelphia protocol IAC 25-000925 and the tissues were placed in 10% neutral-buffered formalin or 4% paraformaldehyde (PFA).

For histology of whole brains, 2- to 5-mo-old mice were perfused transcardially with phosphate-buffered saline (PBS) followed by 4% paraformaldehyde (PFA). Brains were fixed overnight in 4% paraformaldehyde and then placed in 30% sucrose solution.

Histology was performed by HistoWiz Inc. (histowiz.com) using a standard operating procedure and fully automated workflow.

### Mitochondrial Respiration and ROS Production.

Mitochondrial respiration and ROS production were measured using High resolution FluoRespirometry (Oroboros Instruments) and the FluoSensor green for excitation (525 nm) and detection of Amplex Red fluorescence. An air calibration was performed with MiR05 (110 mM sucrose, 60 mM K-lactobionate, 20 mM K-HEPES, 20 mM taurine, 10 mM KH_2_PO_4_, 3 mM MgCl_2_, 0.5 mM EGTA, and 1 mg/mL BSA, pH 7.1) supplemented with 15 µM DTPA (diethylenetriaminepentaacetic acid). Subsequently, Amplex Red (10 µM), horse radish peroxidase (HRP) (1 U/mL) and SOD (2 U/mL) were added, and the fluorescence signal was calibrated by two consecutive additions of H_2_O_2_ (0.2 µM each). Additional ROS calibrations were performed after addition of the sample and at the end of the titration protocol. Mice were killed using cervical dislocation, and brain regions (cortex and cerebellum) were dissected and homogenized in MiR05+DTPA using Dounce homogenizers. Cortex or cerebellum homogenate corresponding to 30 μg protein was added to the respirometry chambers. A substrate-uncoupler-inhibitor titration (SUIT-) protocol was used to evaluate mitochondrial respiration and ROS production at different mitochondrial respiratory states. Titration protocol: pyruvate, malate, and glutamate (5 mM, 0.5 mM, and 10 mM), ADP (5 mM), succinate (10 mM), oligomycin (2.5 µM), FCCP (1.5 µM), rotenone (0.5 µM), and antimycin A (2.5 µM). After each addition, rate of respiration and ROS production were measured. Respiration was corrected for the residual oxygen consumption after antimycin A.

### Mass Spectrometry.

Five flash frozen *Mecr^285/del10^* and five WT mouse cerebella (*n =* 5 for each genotype, all female) were resuspended at 1:5 g:mL in solutions of 100 mM ammonium bicarbonate and 8 M urea and homogenized using an Omni TH. Samples were centrifuged at 20 k×*g* for 5 min at 4 °C. Protein abundance of supernatant was measured by bicinchoninic acid (BCA) assay. One hundred µg of protein was diluted to 50 µL in 100 mM ammonium bicarbonate and adjusted to 10 mM dithiothreitol (DTT) and then incubated at 37 °C for 45 min to reduce cystines to cysteines. Cysteines were alkylated by adding 5.5 µL of 0.5 M iodoacetamide and incubating at RT in a light-free environment for 40 min. Samples were diluted to 150 µL with 50 mM ammonium bicarbonate, and digestion was performed by adding 2 µg of Sequencing Grade Modified Trypsin (Promega) and incubating at 37 °C overnight. Samples were diluted to 0.1% trifluoroacetic acid (TFA) and then desalted using C18 stagetips. Washes were performed with 75 μL of 0.1% formic acid and elution with 100 μL of 0.1% formic acid in 60% acetonitrile (ACN). A Savant SpeedVac was used to dry the samples, which were resuspended in 20 μL of 0.1% TFA. Absorbance at 280 nm was measured using a NanoDrop (Thermo Scientific) to normalize injection volumes. Analysis was performed using a Dionex UltiMate 3000 nanoLC connected to a Q Exactive HF (Thermo Scientific). The LC system was configured with a C18 trap column (Thermo Scientific) and in-house packed C18 (Dr. Maisch, GMBH) analytical column. Buffer A consisted of 0.01% formic acid, and buffer B consisted of 0.01% formic acid in 80% acetonitrile (ACN). The analytical gradient was 5 to 25% buffer B over 90 min followed by 25 to 45% buffer B over 30 min. MS data were acquired using data-independent acquisition (DIA) with 24 m/z MS2 windows. Data processing was performed using DIA-NN with a complete UniProt FASTA digest library-free search without heuristic protein inference (59). Protein abundances were examined without imputation and then imputed for statistical representation.

### Aconitase Activity Assay.

Aconitase activity was determined spectrophotometrically by monitoring the formation of NADPH at 340 nm. The assay mixture contained 50 mM Tris-HCl (pH 7.4), 60 mM sodium citrate, 1 mM MnCl_2_, 20 mM NADP^+^, and 4 units/mL of isocitrate dehydrogenase. 130 µg of the protein extract was brought up to 150 μL with 50 mM Tris-buffer (pH 7.4) and loaded into a 96-well plate. By adding 150 μL of the assay mixture, the enzyme reaction was started, and the change of absorbance at 340 nm was measured for 20 min at 24 °C. The aconitase activity was calculated from the slope of the linear portion.

### SDS–PAGE Immunoblot.

Brain lysates were prepared by resuspending samples in RIPA buffer (50 mM Tris HCl, PH 7.4, 150 mM NaCl, 1% Triton X-100, 0.5% sodium deoxycholate, 0.1% SDS, 1 mM EDTA, and 10 mM Naf) and HALT Protease Inhibitor Cocktail (Thermo Fisher) and homogenizing for 30 s with a Polytron mechanical homogenizer. The samples were solubilized on ice for 20 min and clarified by centrifugation (20,000×*g*, 20 min, 4 °C). Lysate protein content was quantified by Pierce BCA Protein Assay Kit (Thermo Fisher). For each lane, 20 μg of protein was mixed with protein sample loading buffer (Licor) and separated on a NuPAGE 4 to 12% Bis–Tris gel (Thermo Fisher). A prestained Protein Ladder (LI-COR) was used. Resolved proteins were then transferred to PVDF membrane (iBlot 2 Transfer stacks) using the iBlot 2 transfer system. Blots were blocked for 1 h with 5% nonfat dry milk in Tris-buffered saline with Tween-20 (TBST) and probed with primary antibodies for 1 h, followed wash in TBST and 1 h incubation with IRDye-conjugated secondary antibody. Blots were imaged and analyzed using a LI-COR Odyssey M imaging system and LI-COR Empiria Studio software. See *SI Appendix*, Table S1 for detailed antibody information.

### Native PAGE.

Mitochondrial supercomplexes were analyzed using blue native polyacrylamide gel electrophoresis (BN-PAGE) (60). The cerebellum was added to 1 ml mitochondrial isolation buffer in a Dounce homogenizer and disrupted with 15 strokes with a loose pestle and 15 strokes with a tight pestle. The homogenate was centrifuged at 800xg 10 min at 4 °C, and the supernatant was transferred to a fresh tube. The pellet was resuspended in 1 ml MIB and homogenized and centrifuged as before. The supernatants were combined and centrifuged at 8,000×*g* for 10 min at 4 °C. Protein was measured by Bradford Assay (BIO-RAD).

Samples were prepared for native gel electrophoresis using the NativePAGE Sample Prep Kit (ThermoFisher). Mitochondria corresponding to 17ug of protein per lane were resuspended in ice-cold 1X NativePAGE sample buffer containing 1% digitonin. The proteins were solubilized on ice for 15 min and centrifuged at 20,000×*g* for 30 min at 4°C. The supernatant was loaded onto a 4 to 12% NativePAGE gel alongside NativeMark Unstained Protein Standard.

After running for 30 min at 150 V in dark cathode buffer, the cathode buffer was changed to light blue buffer, and the gel was run for an additional 90 min. The gel was removed and soaked in transfer buffer for 15 min before transfer onto PVDF using the iBLOT 2 transfer apparatus. The blot was then blocked in 5% nonfat dry milk TBST for 1 h, incubated with primary antibody for 1 h, and incubated with secondary antibody conjugated to HRP for 1 h. The blots were developed with the ECL kit, visualized using the BIO-RAD ChemiDoc system, and analyzed using ImageJ.

In gel activity assays were performed as described (60).

## Supplementary Material

Appendix 01 (PDF)

## Data Availability

Proteomics data are available at MassIVE with accession number MSV000099206 (61). All other data are included in the article and/or *SI Appendix*.
