# Supplementary material for: A mouse model of MEPAN demonstrates a role for mitochondrial fatty acid synthesis in iron–sulfur cluster and supercomplex formation
Source: Proc Natl Acad Sci U S A. 2025 Sep 29;122(40):e2506761122. doi: 10.1073/pnas.2506761122 (PMC12519216; doi:10.1073/pnas.2506761122)
Supplement: Supplementary file 1 — Appendix 01 (PDF) [file pnas.2506761122.sapp.pdf]

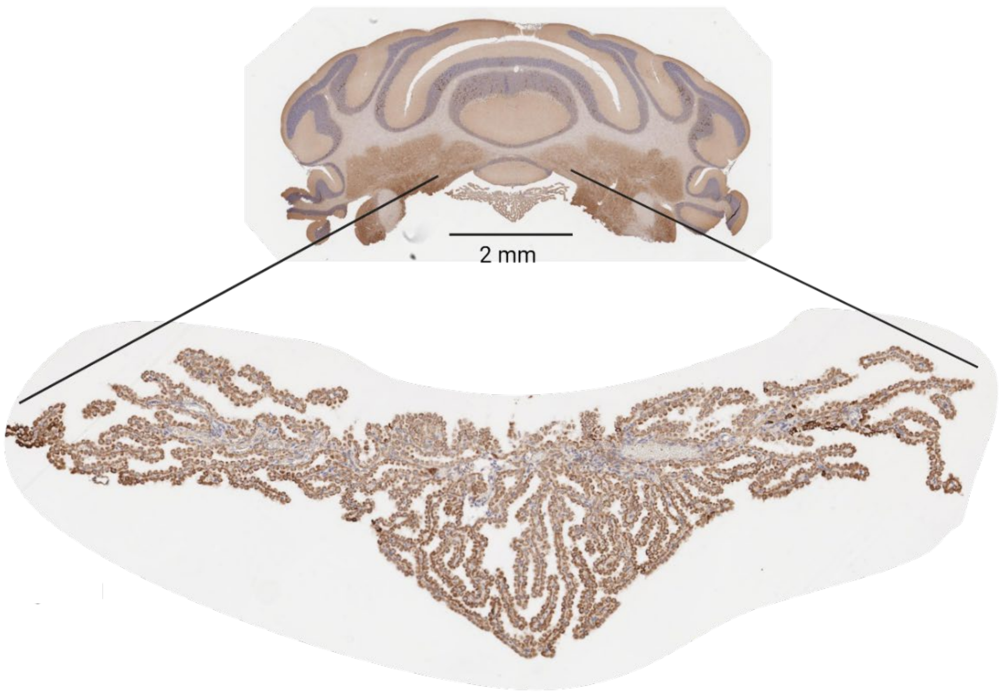

Supplemental Fig S1. Choroid plexus stains with MECR antibody. Sagittal section of the cerebellum of a WT mouse stained with antibodies to MECR. Choroid plexus is shown at a higher magnification, showing strong epithelial layer staining.

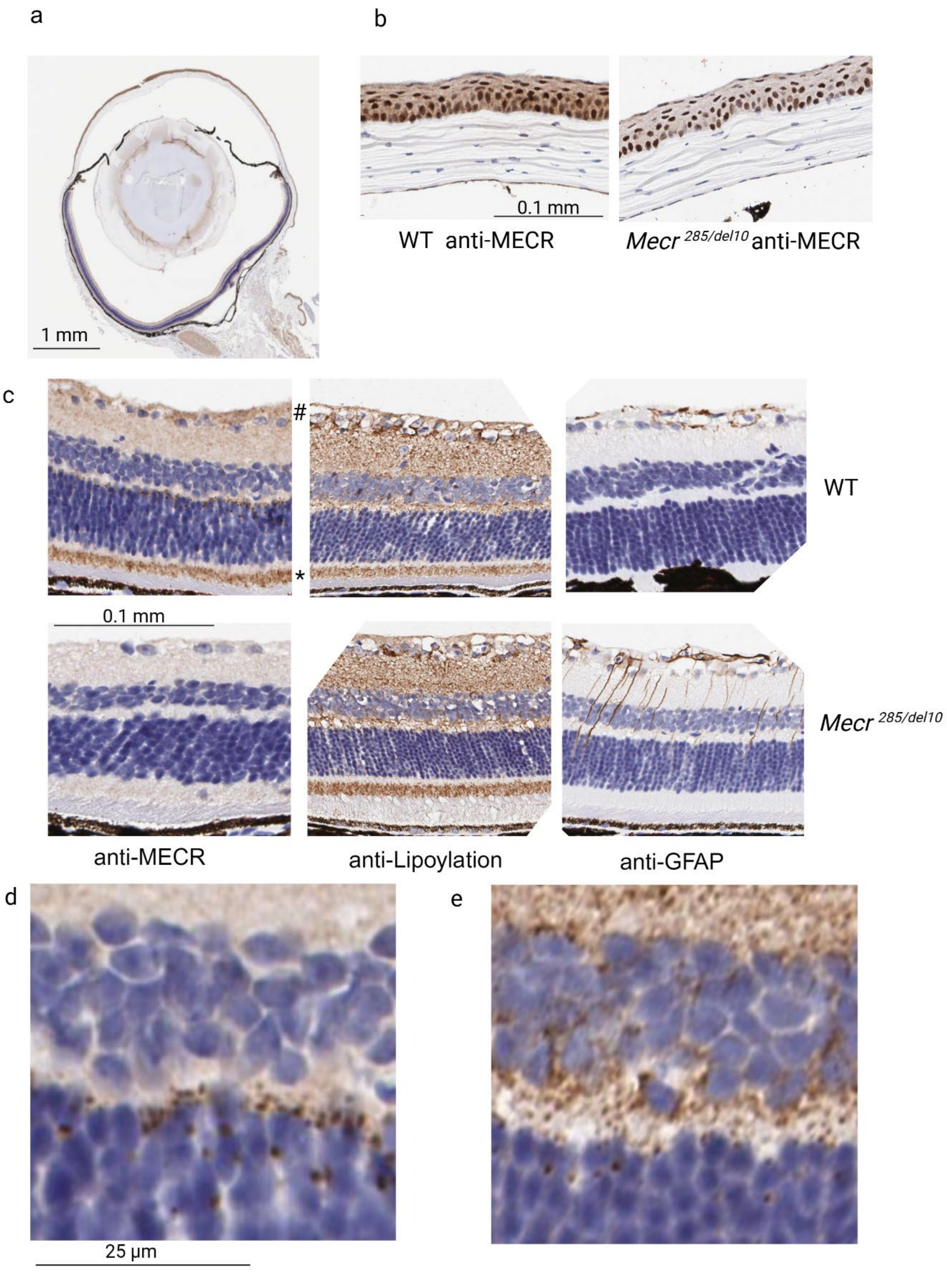
a
b
1 mm
0.1 mm
WT anti-MECR
Mecr 285/del10 anti-MECR
c
#
*
WT
0.1 mm
Mecr 285/del10
anti-MECR
anti-Lipoylation
anti-GFAP
d
e
25 µm

Supplemental Figure S2. Localization of MECR and lipoylated proteins in the WT and *Mecr* $^{285/del10}$ mouse eye. a) Cross section of whole mouse eye stained with MECR antibody, showing staining of cornea and retina. b) Close up of cross section through cornea showing staining with MECR antibody in the squamous cell, wing cell, and basal cell layers. Staining is decreased in the *Mecr* $^{285/del10}$ cornea. c) Close up of MECR antibody and lipoylated protein staining in the retina of WT and *Mecr* $^{285/del10}$ mice MECR staining is concentrated in the elipsoid segment of the photoreceptor cells (*) and in the retinal ganglion cells (#), with diffuse staining of the inner plexiform layer (left column). c) Comparison of staining of retina in WT (top panel) and *Mecr* $^{285/del10}$ (bottom panel) mouse. Bottom left panel shows loss of most MECR antibody staining in the *Mecr* $^{285/del10}$ retina. Lipoylated proteins (middle panels) are localized similarly to MECR staining in the retina, but staining is not lost in the *Mecr* $^{285/del10}$. Right panel shows minimal staining with GFAP antibody in the WT retina which is limited to the nerve fiber layer. *Mecr* $^{285/del10}$ retina (right bottom panel) has increased staining with GFAP antibody through the outer plexiform layer, inner nuclear layer, and inner plexiform layer. d) Close up of MECR antibody staining in the outer plexiform layer shows single large mitochondria adjacent to photoreceptor nuclei inner and outer plexiform layers. (*n* = 3-4 animals per genotype). e) Close up of lipoylated protein antibody staining in the outer plexiform layer shows single large mitochondria adjacent to photoreceptor nuclei inner and outer plexiform layers. (*n* = 3-4 animals per genotype).

a

	Protein	Gene	log2FC	p value	Module
1	NDUV3_MOUSE	Ndufv3	-0.970527	0.0003192	N
2	NDUA8_MOUSE	Ndufa8	-0.983178	0.0003524	ND1
3	NDUS6_MOUSE	Ndufs6	-0.852162	0.0004482	N
4	NDUS4_MOUSE	Ndufs4	-0.886151	0.0007784	N
5	NDUA5_MOUSE	Ndufa5	-0.643898	0.0008766	Q
6	NDUAC_MOUSE	Ndufa12	-0.77087	0.0017919	N
7	NDUA6_MOUSE	Ndufa6	-0.98185	0.0019607	N
8	NDUS3_MOUSE	Ndufs3	-0.558314	0.0031687	Q
9	NDUV2_MOUSE	Ndufv2	-0.800402	0.0032877	N
10	NDUS5_MOUSE	Ndufs5	-0.707939	0.0039603	ND2
11	NDUA7_MOUSE	Ndufa7	-0.939434	0.008581	N
12	NDUF3_MOUSE	Ndufaf3	0.6315139	0.0173249	ACC
13	NDUA2_MOUSE	Ndufa2	-0.793226	0.0199645	N
14	NDUA1_MOUSE	Ndufa1	-1.158732	0.021579	ND2
15	NDUA9_MOUSE	Ndufa9	-0.672415	0.027238	Q
16	NDUS2_MOUSE	Ndufs2	-0.577321	0.0295924	Q
17	NDUV1_MOUSE	Ndufv1	-0.617008	0.0373364	N

b

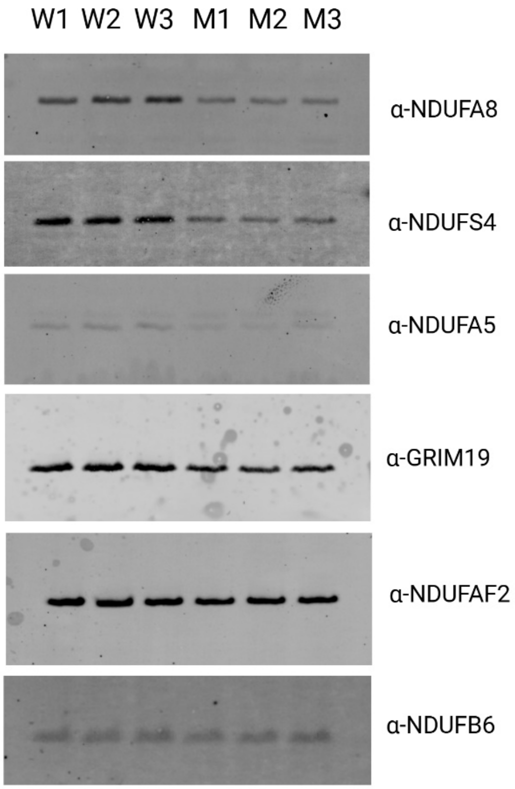

Supplemental Fig S3. Proteins of complex I of the electron transport chain are downregulated in *Mecr*$^{285/del10}$ cerebellum. a) 17 polypeptides of complex I are underrepresented in proteomic analysis of the cerebellum. b) Representative western blots of proteins of complex I found changed in proteomic analysis (*n* = 3 mice per genotype and repeated at least twice).

Supplemental Table S1

Specific crRNAs and Donor Templates used for CRISPR/Cas9 Mutagenesis

**Mutation specific crRNAs**
**Mecr258 crRNA**
/AltR1/rArC rArCrA rGrUrU rGrArG rArGrC rCrArA rUrCrG rGrUrU rUrUrA rGrArG rCrUrA rUrGrC rU/AlrR2/
**Mecr232 crRNA**
/AltR1/rArC rUrGrA rCrArG rArCrU rGrArA rGrGrA rUrCrU rGrUrU rUrUrA rGrArG rCrUrA rUrGrC rU/AlrR2/
**Mecr285 crRNA**
/AltR1/rGrA rGrGrA rArCrC rArUrG rGrUrG rArCrC rUrArU rGrUrU rUrUrA rGrArG rCrUrA rUrGrC rU/AlrR2/
**Donor templates**
**Mecr258 ssODN**
T*A*C* GCT AGG TGC CGG AGC AGC TCT GTA GAA CTC TTC CCA CCA ACA CAG TTG AGA GCC AAC CAG GAC AGC GGC AGG TCC TGA AAG TAA GGA GGC TAC GGT GAG GGG GCT ACT GGG TGC* T*C*A*
**Mecr232 ssODN**
T*TG* T*GT TTG GTT TCC AGA CCC GAC ATC AAG AAG CTA ACT GAC AGA CTG AAG GAT CTA GAG GCT GAT TAT GTC CTC ACA GAG GAA GAG CTA AGG ATG CCC GAG ACA AAA ACC ATC TTC* A*A*G
**Mecr285 ssODN**
T*T*G* CCT GCA CCC ACT CTT CAT TCT CTG AAC TCT ACA GGC CCG GAG GAA CCA TGG TGA CCT GTG GAG GAA TGG CCA AAC AGC CTG TAA CAG CCT CTG TGG TAA GTC AGG GAT ACC TGC*.

Supplemental Table S2. Antibodies used in this study

Antigen	Company	Catalog
ISCU	Proteintech	14812-1-AP
NDUFA7	Thermo Fisher	PA5-37875
NDUFB6	Proteintech	16037-1-AP
NDUFS4	Thermo Fisher	PA5-21677
NDUFV2	Proteintech	15301-1-AP
Lipoic Acid	Millipore Sigma	437695
MTCOI	abcam	ab14705
ATP5A	abcam	ab14748
SDHA	abcam	ab14715
MECR	Proteintech	51027-2-AP
Rabbit anti-Mouse IgG2a HRP	Thermo Fisher	61-0220
Goat anti-Rabbit 800CW IgG	Licor	926-32211
Donkey anti-Mouse 680RD IgG	Licor	926-68072
Rabbit anti-goat IgG HRP	Thermo Fisher	31402
NFS1	Proteintech	15370-1-AP
Goat anti-Rabbit IgG HRP	Jackson Immu	111-035-003
NDUFS3	Proteintech	15066-1-AP
LYRM4	Thermo Fisher	PA5-70317
NDUFAB1	Thermo Fisher	PA5-89967
NDUFA5	Thermo Fisher	PA5-22143
NDUFAB1	NOVUS	NBP3-17896
NDUFS6	Proteintech	14417-1-AP
NDUFA8	Proteintech	15064-1-AP
NDUFV3	Thermo Fisher	PA5-75891
GFAP	NOVUS	NB300-141

Supplemental Table S3
Comparison of Human Phenotypes in Mouse Model of MEPAN

Human MEPAN Phenotype	Mouse MEPAN
Movement disorder	yes, by rotarod and gait analysis
Optic neuropathy	yes, by GFAP expression in retina
Loss of lipoylation of proteins	yes, by Western blot and proteomics analysis
Loss of mitochondrial respiration	yes, by high resolution respirometry of cerebellum
T2 weighted MRI hyperintensity	not tested
